# Involvement of MAP3K8 and miR-17-5p in Poor Virologic Response to Interferon-Based Combination Therapy for Chronic Hepatitis C

**DOI:** 10.1371/journal.pone.0097078

**Published:** 2014-05-12

**Authors:** Akihito Tsubota, Kaoru Mogushi, Hideki Aizaki, Ken Miyaguchi, Keisuke Nagatsuma, Hiroshi Matsudaira, Tatsuya Kushida, Tomomi Furihata, Hiroshi Tanaka, Tomokazu Matsuura

**Affiliations:** 1 Institute of Clinical Medicine and Research (ICMR), Jikei University School of Medicine, Kashiwa, Chiba, Japan; 2 Division of Gastroenterology and Hepatology, Kashiwa Hospital, The Jikei University School of Medicine, Kashiwa, Chiba, Japan; 3 Department of Bioinformatics, Medical Research Institute, Tokyo Medical and Dental University, Bunkyo-ku, Tokyo, Japan; 4 Department of Virology II, National Institute of Infectious Diseases, Shinjuku-ku, Tokyo, Japan; 5 National Bioscience Database Center, Japan Science and Technology Agency, Chiyoda-ku, Tokyo, Japan; 6 Laboratory of Pharmacology and Toxicology, Graduate School of Pharmaceutical Science, Chiba University, Chiba, Japan; 7 Department of Laboratory Medicine, Jikei University School of Medicine, Minato-ku, Tokyo, Japan; Harvard Medical School, United States of America

## Abstract

Despite advances in chronic hepatitis C treatment, a proportion of patients respond poorly to treatment. This study aimed to explore hepatic mRNA and microRNA signatures involved in hepatitis C treatment resistance. Global hepatic mRNA and microRNA expression profiles were compared using microarray data between treatment responses. Quantitative real-time polymerase chain reaction validated the gene signatures from 130 patients who were infected with hepatitis C virus genotype 1b and treated with pegylated interferon-alpha and ribavirin combination therapy. The correlation between mRNA and microRNA was evaluated using *in silico* analysis and *in vitro* siRNA and microRNA inhibition/overexpression experiments. Multivariate regression analysis identified that the independent variables IL28B SNP rs8099917, hsa-miR-122-5p, hsa-miR-17-5p, and MAP3K8 were significantly associated with a poor virologic response. MAP3K8 and miR-17-5p expression were inversely correlated with treatment response. Furthermore, miR-17-5p repressed HCV production by targeting MAP3K8. Collectively, the data suggest that several molecules and the inverse correlation between mRNA and microRNA contributed to a host genetic refractory hepatitis C treatment response.

## Introduction

Chronic hepatitis C (CH-C) caused by hepatitis C virus (HCV) infection is a major chronic liver disease worldwide, and it often develops into cirrhosis and hepatocellular carcinoma. Pegylated interferon alpha (peg-IFNα) and ribavirin (RBV) combination therapy is widely used to treat CH-C [1]. However, treatment fails in approximately 50% patients with HCV genotype 1. Of note, approximately 20–30% patients show null or partial response to the treatment. The introduction of nonstructural 3/4A protease inhibitors has improved the outcome for genotype 1 CH-C patients [1]. However, new antiviral agents increase the frequency and severity of adverse effects, are costly, have complex treatment regimens, and often result in viral resistance. Importantly, the outcomes of triple combination therapy are extremely poor in patients who showed null and partial response to previous peg-IFNα/RBV, compared to treatment-naïve patients and relapsers [1]–[3]. Furthermore, over 50% of null and partial responders, among all patients with a similar virologic response or viral kinetics, relapse after treatment cessation [2], [3]. Collectively, these studies suggest a role of host genetics in treatment resistance.

Microarray applications in clinical medicine identified that numerous mRNAs and microRNAs (miRNAs) regulate complex processes involved in disease development. For example, hepatic mRNA expression of IFN-stimulated genes (ISGs, such as ISG15, OAS, IFI, IP10, and viperin) and IFN-related pathway genes (MX and USP18) correlate with responses to peg-IFNα/RBV combination therapy for CH-C [4]–[7]. However, few studies have examined global miRNAs alone [8]. Furthermore, mRNA and miRNA gene signatures and their interactions in treatment response have not been reported. miRNAs are evolutionarily conserved, small non-coding RNAs [9], [10]. A single miRNA can regulate the expression of multiple target mRNAs and their encoded proteins by imperfect base pairing and subsequent mRNA cleavage/translational repression. Conversely, the expression of a single mRNA is often regulated by several miRNAs. As regulators of promotion or suppression of gene expression, miRNAs are involved in diverse biological and physiological processes, including cell cycle, proliferation, differentiation, and apoptosis. In addition to targeting endogenous mRNAs, miRNAs regulate the life cycle of viruses such as the Epstein-Barr virus, HCV, and other oncogenic viruses by interacting with viral transcripts [11], [12].

We investigated the differential expression profiles of mRNAs and miRNAs isolated from the liver tissues of untreated patients with HCV genotype 1b using microarray analysis. Expression profiles and their interactions were analyzed to identify the molecular signatures associated with treatment resistance.

## Materials and Methods

### Patient population, treatment, and liver tissue samples

During 2010 and 2011, 130 patients infected with HCV genotype 1b were treated weekly with 1.5 µg/kg of peg-IFNα-2b (MSD, Tokyo) and daily with 600–1000 mg RBV (MSD) [2], [3] for 48 weeks at Jikei University Kashiwa-affiliated hospitals. Patients with undetectable serum HCV RNA at week 12 or later were recommended to extend the treatment to 72 weeks. All study participants provided informed written consent and materials for genetic testing and met the following criteria: (1) CH-C diagnosis confirmed by laboratory tests, virology, and histology; (2) genotype 1b confirmed by polymerase chain reaction (PCR)-based method; (3) absence of malignancy, liver failure, or other form of chronic liver disease; and (4) no concurrent treatment with any other antiviral or immunomodulatory agent. Liver specimens were obtained percutaneously before treatment, formalin-fixed, and paraffin-embedded for histological assessment [13]. A tissue section was stored in RNA*later* solution (Life Technologies, Carlsbad, CA). Total RNA containing mRNA and miRNA was isolated using the mirVana miRNA isolation kit (Life Technologies).

Sustained virological response (SVR) was defined as an undetectable serum HCV RNA level at 24 weeks after treatment completion. A null response was defined as a viral decline of <2 log_10_ IU/mL from baseline at treatment week 12 and detectable HCV RNA during treatment. A partial response was defined as a viral decline of >2 log_10_ IU/mL from baseline at week 12, with no achievement of an undetectable HCV RNA level. Relapse was defined as an undetectable serum HCV RNA level at the end of treatment and viremia reappearance on follow-up examination [1]. Viral loads and the presence or absence of serum HCV RNA were evaluated using a qualitative PCR assay (Amplicor HCV version 2.0; Roche Diagnostics, Tokyo).

This study conformed to the provisions of the Declaration of Helsinki and Good Clinical Practice guidelines and was approved by the Jikei University Ethics Committee for Human Genome/Gene Analysis Research (No.21-093_5671).

### mRNA microarray

Global mRNA expression analysis was performed using total RNA isolated from each sample [sustained virological responders (SVRs), *n* = 5; relapsers, *n* = 3; null responders, *n* = 4] and the GeneChip Human Genome U133 Plus 2.0 Array (Affymetrix, Santa Clara, CA). Datasets were normalized by the robust multi-array analysis, using R 2.12.1 statistical software and the BioConductor package.

### miRNA microarray

Global miRNA expression analysis was performed using total RNA isolated from the same samples used for mRNA expression analysis and the miRCURY LNA microRNA Array series (Exiqon, Vedbaek, Denmark). Total RNA was labeled with Hy3 and hybridized to slides that contained capture probes targeting all human miRNAs registered in the miRBASE 14.0. miRNA microarray datasets were normalized by quantile normalization using R statistical software.

### Differential gene expression according to treatment response

The limma package from BioConductor software (under R statistical software) was used to calculate moderated t-statistics (based on the empirical Bayes approach) to identify mRNA or miRNA differentially expressed between the SVR/relapser group and null/partial responder group. Because of multiple hypothesis testing, *p* values were adjusted by the Benjamini-Hochberg false discovery rate (FDR) method.

### Hierarchical cluster analysis

Up- and down-regulated probe sets were analyzed by hierarchical clustering using R statistical software. Pearson's correlation coefficients were used to calculate a matrix similarity score among the probe sets. The complete linkage method was used for agglomeration. Heat maps were generated from significant differentially expressed probe sets.

### Quantitative real-time PCR for mRNA

To validate microarray results and to confirm the observed differences in the mRNA expression levels in a quantitative manner, each sample was subjected to reverse transcription (RT)-PCR and quantitative real-time RT-PCR (qPCR) in triplicate. After cDNA synthesis, target genes were amplified in PCR mixtures that contained TaqMan Universal PCR Master Mix (Life Technologies) and TaqMan probes designed with the Universal Probe Library Assay Design Center (http://www.roche-applied-science.com/sis/rtpcr/upl/adc.jsp). Target gene expression levels in each sample were normalized to the expression of the housekeeping gene of 18S rRNA and the corresponding gene of one null responder.

### Quantitative real-time PCR for miRNA

cDNA was synthesized from aliquots of the isolated total RNA using the TaqMan MicroRNA Reverse Transcription kit (Life Technologies) including RT primers designed with miRNA-specific stem-loop structures according to manufacturer's protocol. miRNA expression levels were quantified with the TaqMan MicroRNA assay (Life Technologies) in triplicate. Target gene expression levels were normalized in each sample to the expression of the endogenous gene RNU48 and the corresponding gene of one null responder.

### miRNA target prediction

Up- and down-regulated miRNAs with a fold change of >1.2 and *p*<0.005 (FDR<0.15) between two groups (SVRs/relapsers *vs* null responders) in the microarray analysis were subjected to the *in silico* prediction of mRNA targets for miRNA using MicroCosm Targets, miRanda, PicTar, PITA, and TargetScan algorithms. Predicted mRNA targets were analyzed further if they met the following criteria: (1) fold change of >1.5 and *p*<0.003 (FDR<0.35) in mRNA microarray results; (2) inverse correlation (negative correlation coefficient) between miRNA and mRNA in mRNA and miRNA microarray results; and (3) qPCR-validated microarray results. Kyoto Encyclopedia of Genes and Genomes (KEGG) Pathways, Agilent Literature Search 3.0.3 beta, and Cytoscape 3.0.2 were used to identify the significance of candidates in gene regulatory networks.

### Cell culture

The human hepatoma cell line Huh7.5.1 (a gift from Professor Francis Chisari, Scripps Research Institute, La Jolla, CA) was maintained in Dulbecco's modified Eagle's medium containing 10% fetal bovine serum [14]. Cell culture-produced HCV (HCVcc) were harvested from JFH1-transfected Huh7.5.1 as previously described [15].

### Plasmids and siRNAs

The siRNAs targeting MAP3K8 were siRNA1, 5′-uucgucuuuauaucuugugtt-3′; siRNA2, 5′-uguugcuagguuuaauauctt-3′; siRNA3, 5′-aucuugugccaaguauacctt-3′; and scrambled negative control siRNA to siRNA1 (Sigma-Aldrich, St. Louis, MO). The expression and inhibitor plasmids of hsa-miR-17-5p and control plasmids were purchased from GeneCopoeia (Rockville, MD).

### HCV core antigen measurements and cell viability

The HCV core antigen concentrations in filtered culture medium and cell lysates of infected cells were measured with the Lumipulse Ortho HCV antigen kit (Ortho Clinical Diagnostics, Tokyo). Cell viability was analyzed using the CellTiter-Glo Luminescent Cell Viability Assay (Promega, Madison, WI).

### Transfection

Cells were seeded into a 24-well plate and transfected with siRNAs and plasmids using Lipofectamine RNAiMAX (Invitrogen, San Diego, CA) and TransIT-LT1 (Mirus, Madison, WI), respectively.

### Luciferase reporter assay

The MAP3K8 3′UTR segment containing the putative miR-17-5p target sites was subcloned into the pGL3 reporter plasmid (Promega). A mutant construct was also generated by PCR-based mutagenesis using mutagenic primers. The luciferase reporter and hsa-miR-17-5p expressing or mock plasmids were co-transfected with the *Renilla* luciferase transfection control plasmid. Luciferase reporter activity was measured 48 hours after transfection with the Dual-Luciferase Reporter Assay System (Promega).

### Western blot

Liver samples were sonicated in lysis buffer. Lysate aliquots were separated by SDS-polyacrylamide gel electrophoresis and transferred onto a nitrocellulose membrane. Membranes were incubated with primary antibodies against MAP3K8 (ab70853, Abcam, San Diego, CA) and β-actin (EP1123Y, Abcam). Membranes were incubated with horseradish peroxidase-conjugated secondary antibodies. Immunoreactivity was detected with reagents (GE Healthcare Life Sciences, Piscataway, NJ). Images were scanned and band intensities quantified with Image J.

### IL28B and ITPA single nucleotide polymorphism (SNP) genotyping

Genomic DNA was extracted from whole blood using the MagNA Pure LC and the DNA Isolation Kit (Roche Diagnostics). The IL28B rs8099917 and rs12979860 [16], [17] and ITPA exon 2 rs1127354 [18] genetic polymorphisms were genotyped by real-time detection PCR with the TaqMan SNP Genotyping Assays.

### Statistical analysis for factors associated with null/partial response

The chi-square, Fisher's exact, Student's *t*, and the Mann–Whitney two-tailed tests were used to compare frequencies in categorical data or differences in continuous data between two groups. Significant independent factors associated with null/partial responses were identify with multiple logistic regression analysis using the SPSS statistical package for Windows, version 17.0 (IBM SPSS, Chicago, IL). A *p* value of <0.05 was considered statistically significant.

## Results

### Patient profiles and treatment response

Among the 130 patients, 62 (48%) achieved SVR, 36 (28%) relapsed, and six (5%) and 26 (20%) showed partial and null response, respectively. Patients were divided into an SVR/relapser group and a null/partial responder group. Table S1 compares the baseline characteristics of the two groups. Patients with elevated serum gamma glutamyl transpeptidase and decreased albumin concentrations were more likely to experience a null/partial response. The IL28B rs8099917 TG and rs12979860 CT variants were more likely to be null/partial responders compared with TT and CC genotypes, respectively.

### mRNAs associated with treatment response

**Figure 1:**
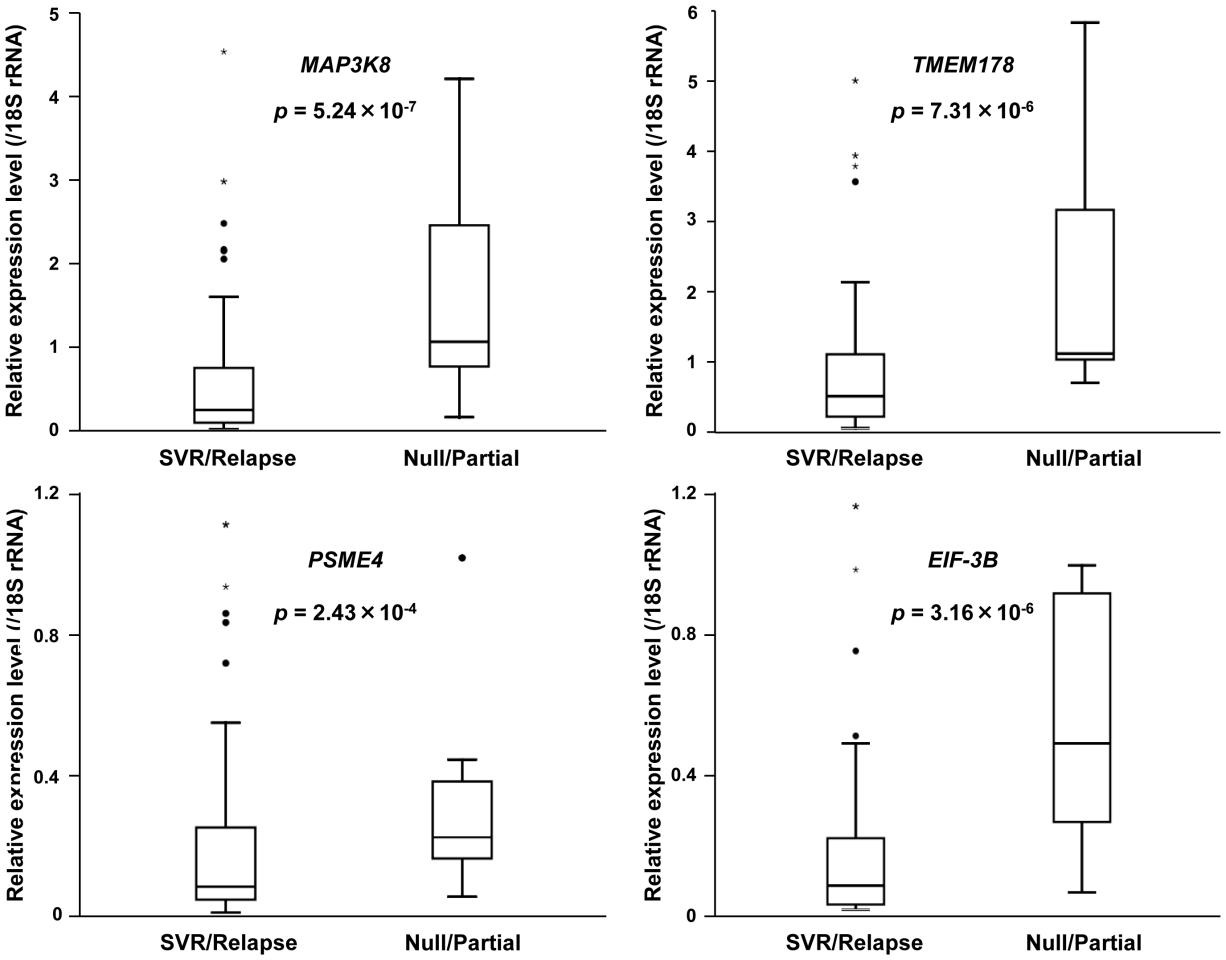
Validation of differentially expressed mRNAs by qPCR analysis. The expression levels of four mRNAs were significantly higher in null/partial responders than in SVRs/relapsers. Assays for each sample were performed in triplicate. All *p*-values were calculated using the Mann–Whitney test.

mRNA microarray data were deposited into the NCBI Gene Expression Omnibus (GEO) (http://www.ncbi.nlm.nih.gov/geo/), accession number GSE42697. The cut-off criteria for fold change >1.5 and *p*<0.003 identified 39 up-regulated and 17 down-regulated annotated probe sets in null responders (Data S1). The up-regulated genes were associated with transcription, translation, cell cycle, phosphorylation, signal transduction, immune response, RNA splicing/mRNA processing, and viral reproduction. The down-regulated genes were associated with xenobiotic/small molecule/lipid metabolic and oxidation–reduction processes. Hierarchical clustering of mRNAs and samples showed that samples from SVRs and relapsers clustered to form a group different from the null responders (Fig. S1). To validate the microarray results, qPCR was performed for all significant differentially expressed genes. The expression levels of MAP3K8 (mitogen-activated protein kinase kinase kinase 8, *p* = 5.24×10^−7^), TMEM178 (transmembrane protein 178, *p* = 7.31×10^−6^), PSME4 (proteasome activator subunit 4, also known as *PA200*, *p* = 2.43×10^−4^), and EIF3B (eukaryotic translation initiation factor-3B, *p* = 3.16×10^−6^; Fig. 1) were significantly increased in null/partial responders compared with those in SVRs/relapsers. TMEM178 is a multi-pass membrane protein and PSME4 is a nuclear protein that activates the proteasome and is important for oxidative-stress adaptation. EIF3B is involved in protein translation/synthesis and interacts with the HCV IRES and the 40S ribosomal subunit.

### miRNAs associated with treatment response

**Figure 2:**
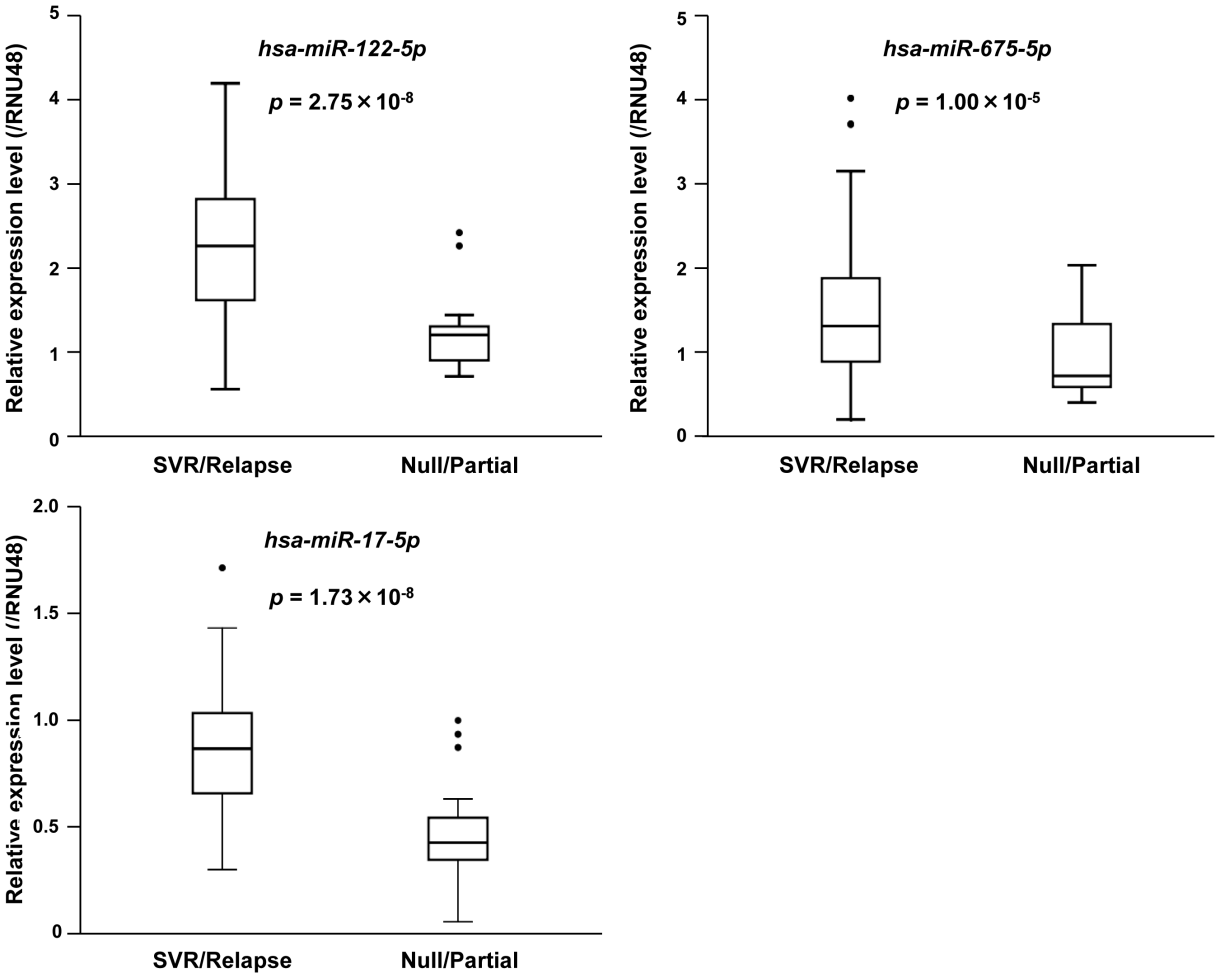
Validation of differentially expressed miRNAs by qPCR analysis. The expression levels of three miRNAs were significantly higher in null/partial responders than in SVRs/relapsers. Assays for each sample were performed in triplicate. All *p*-values were calculated using the Mann–Whitney test.

miRNA microarray data were deposited into the NCBI GEO, accession number GSE45179. The cut-off criteria for fold change >1.2 and *p*<0.005 identified 111 down-regulated and 76 up-regulated miRNAs in null responders (Data S2). Hierarchical two-dimensional clustering showed that distinct patient groups clustered into two distinct groups (Fig. S2). Bioinformatic analysis predicted target genes of the differentially expressed miRNAs. The hypothetical target genes should be MAP3K8, TMEM178, PSME4, and EIF3B. Furthermore, these miRNAs and mRNAs must have an inverse correlation between mRNA and miRNA microarray data. The microRNAs that satisfied the requirements were as follows: hsa-let-7g* and hsa-miR-17-5p, -20b, -297, -374b, -494, -602, -668, and -1297 for MAP3K8; hsa-miR-106b* and -122-5p for TMEM178; and hsa-miR-492 and -675-5p for PSME4. No corresponding miRNA was identified for EIF3B. Stem-loop-based qPCR was performed to confirm the reliability of the miRNA microarray results and the inverse correlation between miRNA and mRNA. The expression levels of hsa-miR-122-5p (*p* = 2.75×10^−8^), hsa-miR-675-5p (*p* = 1.00×10^−5^), and hsa-miR-17-5p (*p* = 1.73×10^−8^) were significantly lower in null/partial responders than in SVRs/relapsers (Fig. 2).

### Independent variables associated with treatment response

Multiple logistic regression analysis of variables that were significant in univariate analysis identified that rs8099917 [*p* = 3.67×10^−3^, odds ratio (OR) = 7.51, 95% confidence interval (CI) = 2.14–29.27], hsa-miR-122-5p (*p* = 5.60×10^−4^, OR = 0.11, 95% CI = 0.03–0.38), hsa-miR-17-5p (*p* = 2.02×10^−4^, OR = 0.56, 95% CI = 0.41–0.76), and MAP3K8 (*p* = 8.58×10^−3^, OR = 2.86, 95% CI = 1.31–6.25) were significantly associated with null/partial response. Importantly, *in silico* analysis and microarray data suggested that increased miR-17-5p could cause MAP3K8 reduction. In fact, an inverse correlation was observed between MAP3K8 mRNA and miR-17-5p (*r* = −0.592, *p* = 4.31×10^−3^). MAP3K8 is closely linked to genes associated with cell proliferation, inflammation, and apoptosis (Fig. S3) and is associated with the miR-17 cluster family (Fig. S4).

### MAP3K8 contributes to HCV production

**Figure 3:**
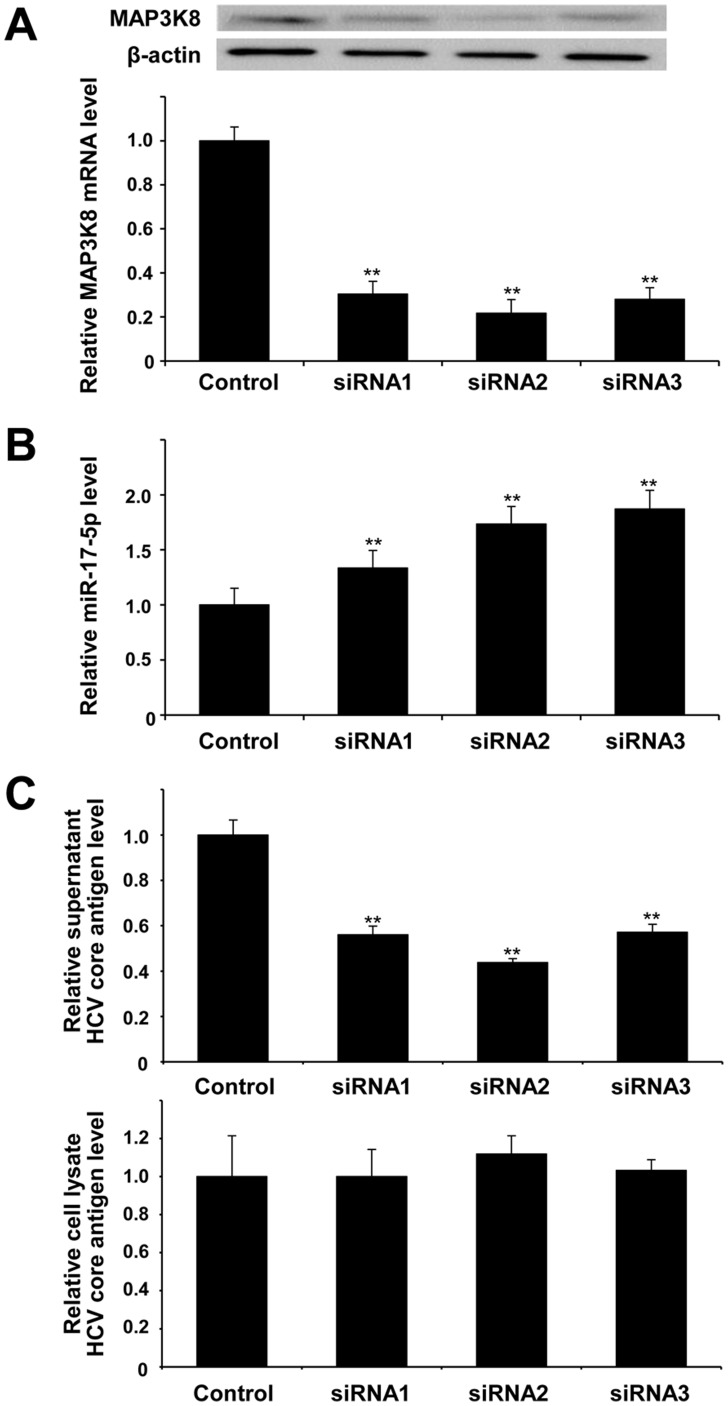
Transfection of Huh7.5.1 cells with siRNAs against MAP3K8. (**A**) Transfection of Huh7.5.1 cells with siRNAs against MAP3K8 significantly decreased intracellular MAP3K8 mRNA levels, (**B**) increased intracellular hsa-miR-17-5p levels, and (**C**) decreased HCV core antigen levels in the supernatant, and had no effect on those in cell lysate. Bars indicate the means of three independent experiments and the error bars indicate standard deviations. All *p*-values were calculated using two-tailed Student's *t*-test. **p<0.001 compared with controls.

siRNA transfection in HCVcc-infected cells was performed to assess the influence of MAP3K8 mRNA and protein on HCV production (Fig. 3A). miR-17-5p levels were significantly increased (Fig. 3B) while supernatant HCV core antigen levels were significantly decreased following transfection of the siRNAs (Fig. 3C). However, the HCV core antigen levels in cell lysates were not changed (Fig. 3C). Taken together, these findings suggested that MAP3K8 repressed miR-17-5p and contributed to the production (e.g. release and assembly) of HCV. *In vivo*, MAP3K8 protein expression levels were significantly increased in null/partial responders compared with those in SVRs/relapsers (*p* = 2.43×10^−5^).

### Hsa-miR-17-5p regulates HCV production by targeting MAP3K8

**Figure 4:**
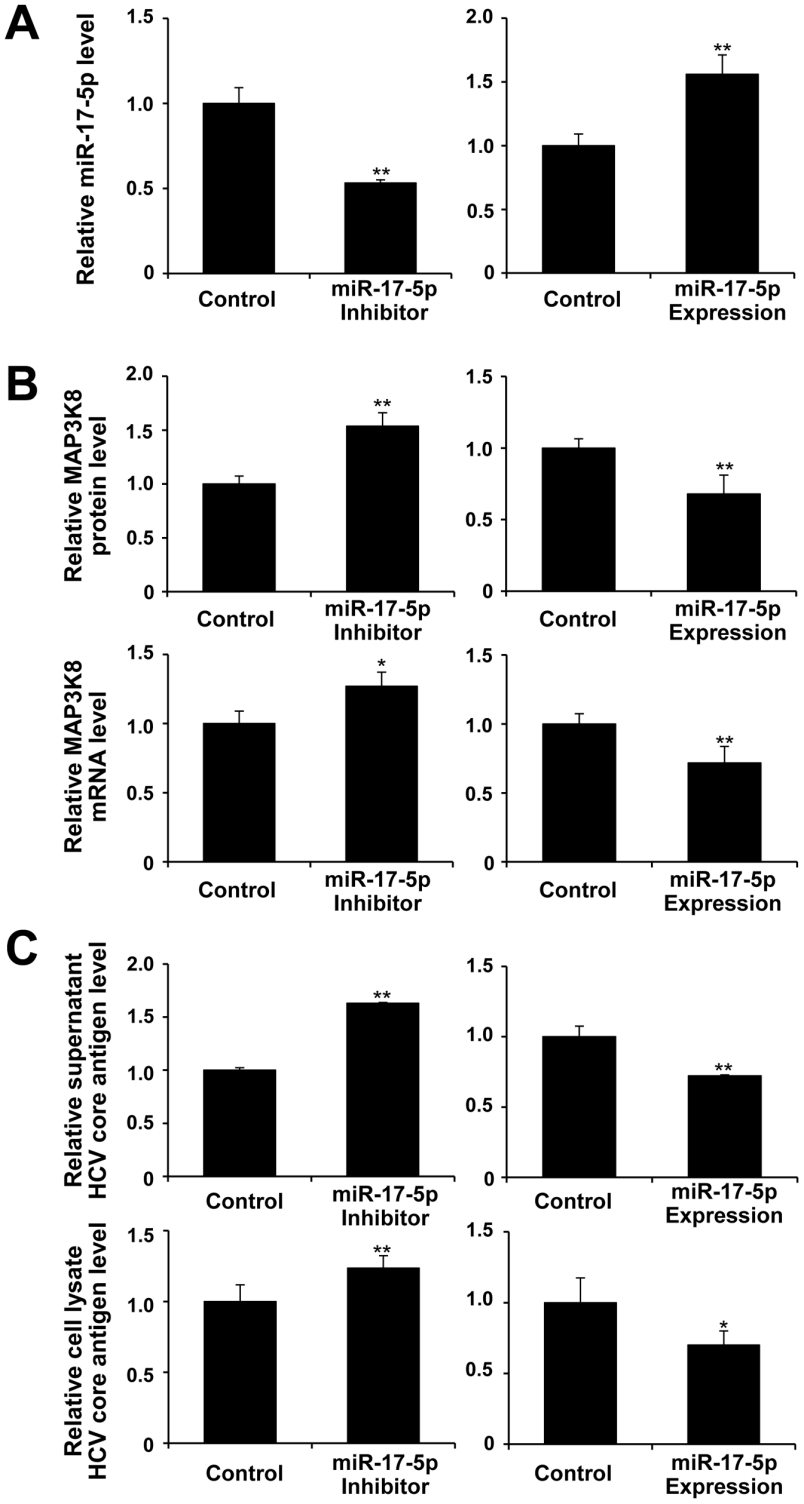
Expression and inhibition of hsa-miR-17-5p in Huh7.5.1 cells. (**A**) Functional suppression (left) and overexpression (right) plasmids of miR-17-5p. (**B**) Inhibition of miR-17-5p increased (left), whereas overexpression of miR-17-5p decreased MAP3K8 mRNA and protein expression levels (right). (**C**) HCV core antigen levels increased following miR-17-5p inhibition (left) and decreased by miR-17-5p overexpression (right) in both supernatant and cell lysate. Bars indicate the means of three independent experiments and the error bars indicate standard deviations. *p<0.01 and **p<0.001 compared with controls.

**Figure 5:**
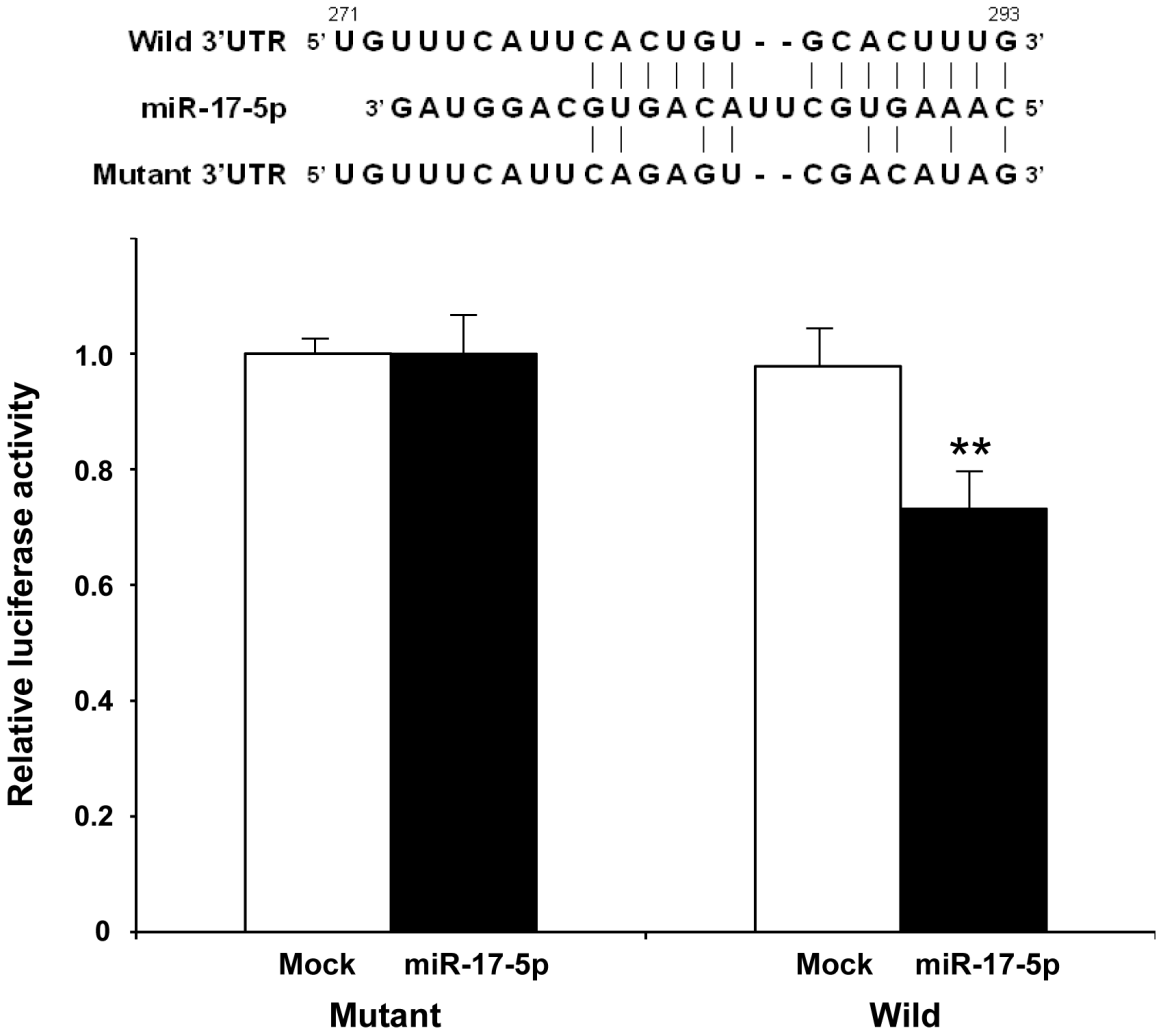
Luciferase reporter assay. miR-17-5p inhibited the luciferase activity of the wild-type MAP3K8 3′UTR construct (right), whereas no decrease in activity was observed in cells co-transfected with the mutant MAP3K8 3′UTR construct (left) or mock plus wild-type or mutant construct (right and left, respectively). Bars indicate the means of three independent experiments, and the error bars indicate standard deviations. **p<0.001 compared with controls.

Changes in MAP3K8 and HCV core antigen levels were evaluated by hsa-miR-17-5p inhibition and overexpression in HCVcc-infected cells. miR-17-5p inhibition increased MAP3K8 mRNA and protein levels (Fig. 4A and 4B, left). In contrast, miR-17-5p overexpression decreased MAP3K8 mRNA and protein levels (Fig. 4A and 4B, right). Interestingly, miR-17-5p inhibition increased, whereas miR-17-5p overexpression decreased HCV core antigen levels in both supernatants and cell lysates (Fig. 4C). Taken together, these results suggested that miR-17-5p regulated the production of HCV by targeting MAP3K8 mRNA. Luciferase reporter assays showed that miR-17-5p overexpression decreased the luciferase activity of the wild-type MAP3K8 3′UTR reporter construct, whereas co-transfection with the mutant MAP3K8 3′UTR construct or mock had no effect (Fig. 5), suggesting that miR-17-5p targeted the MAP3K8 3′UTR and antagonized MAP3K8 protein expression.

## Discussion

This study showed close linkage between mRNA and miRNA signatures in CH-C treatment outcomes using global expression profiling analyses. To confirm the findings, this cohort was randomly divided into derivation and confirmatory groups. The derivation group results were similar to those described above and reproducible in the confirmatory group (data not shown). Subsequently, we attempted to compare our findings with registered patient data obtained from independent cohorts comprising either Asian or non-Asian subjects. However, comparisons were not possible because most mRNA or miRNA microarray studies had a small sample size, limited information, unregistered data, and/or findings that were not validated in an independent cohort [4]–[7], [11], [19]–[21]. To our knowledge, our study was the first to investigate the correlation between mRNA and miRNA in treatment response using global gene expression analysis and *in vitro* experiments. Such gene signature identification can improve the accuracy of treatment outcome predictions, independent of known strong predictors.

Pretreatment hepatic ISG levels are higher in non-SVRs/non-relapsers than in SVRs/relapsers [4]–[7]. The poor ISG response of non-SVRs with further exogenous IFN may contribute to treatment failure [5], [6]. Because patient groups with different response categories differ in their innate IFN response to HCV infection; poor responders may have adopted a different equilibrium in their innate immune response to HCV [4], [6]. As per multivariate regression analysis, however, IL28B SNPs may diminish the significance of hepatic ISGs as treatment predictors because hepatic ISG expression is associated with IL28B SNPs [7], [19]. Conversely, hepatic ISGs were reported to be stronger predictors compared with IL28B SNPs [20]. Although our gene set enrichment analysis (data not shown) also showed that hepatic ISG expression levels were generally higher in null/partial responders than in SVRs/relapsers, the differences were not large enough to be ranked in a higher order and/or to reach statistical significance in expression profiling and validation analyses (Data S3). These variations among studies may be caused by different and heterogeneous patient characteristics, including HCV genotype, patient race, treatment response definitions, study end-points, and treatment regimens. This study analyzed patients with a homogeneous race and genotype (1b) who adhered to combination therapy and treatment for a specified duration.

MAP3K8, also known as cancer Osaka thyroid (cot) [22] or tumor progression locus 2 (tpl2) [23], was originally recognized as a proto-oncogenic protein. Toll-like receptors (TLRs) are innate immune sensors stimulated by specific microbial and viral components, including HCV. *In vitro* HCV infection directly induces TLR4 expression and activates human B cells to increase the production of IFN-β and IL-6 [24]. Peripheral blood mononuclear cells from HCV-infected individuals express higher TLR4 levels compared with uninfected controls [24]. In the IKK-NF-κB pathway, certain activated TLRs, including TLR4, induce inhibition of kappa B kinase (IKK)- catalyzed phosphorylation of nuclear factor kappa B (NF-κB) p105. Nonphosphorylated NF-κB p105 forms a stable, inactive complex with MAP3K8. Subsequent ubiquitination and proteasome-mediated processing of NF-κB-p105 to NF-κB-p50 releases MAP3K8, which activates the MAPK/ERK kinase (MEK)-extracellular signal-regulated kinase (ERK) pathway. MEK-ERK regulates the expression of pro- and anti-inflammatory mediators that lead to the production of various cytokines and chemokines in a stimulus- and cell/receptor type-specific manner (Fig. S3, Fig. S4) [25], [26]. Indeed, MAP3K8 is an important and novel therapeutic target for inflammatory diseases [27]. MAP3K8 is involved in ERK signaling activation in hepatic Kupffer and stellate cells with being stimulated by TLR4 and TLR9, leading to ERK-dependent expression of the fibrogenic genes IL-1β and TIMP-1. Thus, MAP3K8 expression may contribute to liver fibrosis [28].

In addition, this study provided a novel insight into MAP3K8, which is involved in resistance to HCV treatment. The results of experiments in this study demonstrated the importance of MAP3K8 in HCV production. MAP3K8 knockdown by siRNA altered extracellular, but not intracellular, HCV core antigen levels. This result suggests that MAP3K8 might be involved in the release or assembly of HCV, does not exclude the possibility that MAP3K8 participates in intracellular HCV core production because miR-17-5p influenced both supernatant and cell-lysate HCV core antigen levels along with MAP3K8 mRNA and protein levels. If MAP3K8 limited viral release/assembly alone, intracellular HCV core antigen would accumulate following siRNA transfection. Conversely, MAP3K8 overexpression did not affect HCV production, probably because enough MAP3K8 may exist in the cells. This result is generally observed in other critical host factors (e.g. hVAP-33) involved in the HCV life cycle [29]. The above description [24]–[26] and *in silico* analyses (Fig. S3, Fig. S4) suggest that MAP3K8 might play a role in HCV production through a regulatory pathway and network (Fig. S5); however, the exact mechanism remains unknown and requires further investigation. It is important to note that there may be differences between the HCV genotype 1b- and 2a-derived strains/replicons. The 2a-derived JFH1 infection system is highly competent compared with other genotype-derived systems and allows steady inhibition and expression analyses [30]. Notably, this *in vitro* study focused on the correlation between MAP3K8 and miR-17-5p and their impact on HCV production; there may not be significant genotypic effect on MAP3K8 and miR-17-5p. Importantly, it is difficult to determine genotype-specific differences using different infection-competent systems.

The miR-17-92 polycistron, also known as the first oncomir, encodes six or seven miRNAs, including miR-17-5p [31], [32], and is frequently overexpressed in several tumors [31], [33]. In contrast, overexpression of miR-17-5p also leads to tumor suppression in breast cancer [34] and HeLa cells [32]. miR-17-5p may function as both a tumor suppressor and an oncogenic activator by targeting both pro- and anti-proliferative genes and by competing with each other in different cellular contexts, which are dependent on the expression of other transcriptional regulators [35]. Known targets of the miR-17-92 cluster primarily regulate cell cycle progression, apoptosis, and transcription factors [32], [35]. Physiologically, this cluster is down-regulated during aging, and hematopoietic and lung differentiation. During HIV infection, suppression of this cluster by the virus is required for efficient viral replication [36]. Our results suggest that inhibition of miR-17-5p expression may be advantageous for HCV production. Interestingly, miR-17-5p overexpression in HeLa cells decreases the expression of the low-density lipoprotein (LDL) receptor (LDLR) and consequently induces reduced intracellular lipoprotein accumulation because of the impaired internalization [32]. LDLR is one of putative HCV receptors; however, its precise role remains controversial [37]-[40]. LDLR also aids the optimization of HCV replication, and the expression levels are stimulated by HCV infection. Decreased LDLR and lipoprotein uptake through LDLR may adversely affect the HCV life cycle because hepatocyte lipid metabolism pathways are required for HCV.

Bioinformatics and *in vitro* experiments showed that miR-17-5p expression levels were inversely correlated with MAP3K8 in response to anti-HCV treatment. miR-17-5p repressed HCV production by inhibiting MAP3K8 expression, whereas miR-17-5p expression was influenced by MAP3K8. The results also suggested a specific interaction between miR-17-5p and MAP3K8 3′UTR, which was previously validated by the luciferase reporter assay [35]. Taken together, MAP3K8 expression following HCV infection is negatively influenced by miR-17-5p at both the translational and transcriptional levels. This molecular interaction is a potential target for novel molecular therapeutics. However, a single miRNA can regulate the expression of multiple target mRNAs by imperfect base pairing [9], [10]. Conversely, the expression of a single mRNA may be regulated by several miRNAs. Numerous mRNAs and miRNAs are key regulators in complicated pathophysiological networks (Fig. S3, Fig. S4). Therefore, it is important to note that the complex interaction between MAP3K8 and miR-17-5p may not be reflective of a correlation between their expression and viral load in our patient cohort.

Abundant hepatic miR-122 expression is essential for efficient HCV replication in cultured human hepatoma cells [11]. Suppression of miR-122 leads to a marked reduction and long-lasting suppression of HCV RNA in both sera and the livers of nonhuman primates with chronic HCV infection [41]. Paradoxically, this study showed significantly lower miRNA-122 (hsa-miR-122-5p) expression levels in null/partial responders than in SVRs/relapsers, independent of other factors. This finding is in agreement with the results of a previous study, which reported markedly low baseline miR-122 levels in poor responders [21]. Moreover, no positive correlation was observed between miR-122 expression and viral load. No convincing explanation exists for these paradoxical results. Re-analysis of registered miRNA microarray data [8] identified significantly low miR-122 levels, no change in miR-675-5p levels, and low (although not significant) miR-17-5p levels in null/partial responders. Most miR-122 target genes are involved in the lipid biogenesis pathway [42], and miR-122 antagonism induces a substantial decrease in plasma lipid levels. As described above, host lipid metabolism is vital to HCV [40], and may be related to the endogenous IFN response to HCV and IL28B SNPs [43]. However, we did not find a correlation between miR-122 expression and serum lipid levels nor identify miR-122 target genes, including lipid-related metabolic pathways, which could be considered key molecular signatures contributing to a null/partial response.

In conclusion, both global mRNA and miRNA expression profiling analyses increase our understanding of the molecular mechanisms that underlie refractory treatment responses and are even applicable to next-generation treatment. The results obtained in this study also aid the identification of novel features of known genes and target molecules for future therapeutic intervention.

## Supporting Information

Figure S1 **Hierarchical cluster analysis of mRNA expression using microarray analysis.** Changes in mRNA expression levels are presented in graduated color patches from green (least expression) to red (most abundant expression).
(TIF)

Figure S2 **Hierarchical cluster analysis of miRNA expression using microarray analysis.** Changes in gene expression are presented in graduated color patches from blue (least expression) to red (most abundant expression).
(TIF)

Figure S3 **Relationship between MAP3K8 (Tpl2/Cot) and related genes in underlying gene regulatory networks.** MAP3K8 (Tpl2/Cot) was integrated by Kyoto Encyclopedia of Genes and Genomes (KEGG) Pathways. MAP3K8 (Tpl2/Cot) was identified as an important node and considered to be a key regulator.
(TIF)

Figure S4 **Gene networks for MAP3K8 and hsa-miR-17.** MAP3K8 and hsa-miR-17 and array-independent/literature-based text-mining were integrated into the gene regulatory network analysis (Agilent Literature Search). The interaction data were visualized and analyzed by Cytoscape. MAP3K8 and its related mRNAs were associated with the miR-17 cluster family and its related miRNAs via IRF6, STAT3, AKT1, EPHB2, TIMP1, and VEGFA.
(TIF)

Figure S5 **Postulated scheme for HCV replication regulated by MAP3K8 and hsa-miR-17-5p.** IKK, inhibition of kappa B kinase; NF-κB, nuclear factor kappa B; MAP3K8, mitogen-activated protein kinase kinase kinase 8; MEK, MAPK/extracellular signal-regulated kinase.
(TIF)

Table S1 **Comparison of baseline profiles between SVRs/relapsers and null/partial responders.**
(DOC)

Data S1 **List of gene probe sets up- and down-regulated in sustained virological responders (SVR) and relapsers compared with those in null responders.**
(XLS)

Data S2 **List of microRNA probe sets up- and down-regulated in sustained virological responders (SVR) and relapsers compared with those in null responders.**
(XLS)

Data S3 **List of all gene probe sets up- and down-regulated in sustained virological responders (SVR) and relapsers compared with those in null responders, and gene signatures in previously reported references.**
(XLS)
