# Supplementary figures and images for: Involvement of MAP3K8 and miR-17-5p in Poor Virologic Response to Interferon-Based Combination Therapy for Chronic Hepatitis C
Source: PLoS One. 2014 May 12;9(5):e97078. doi: 10.1371/journal.pone.0097078 (PMC4018277; doi:10.1371/journal.pone.0097078)

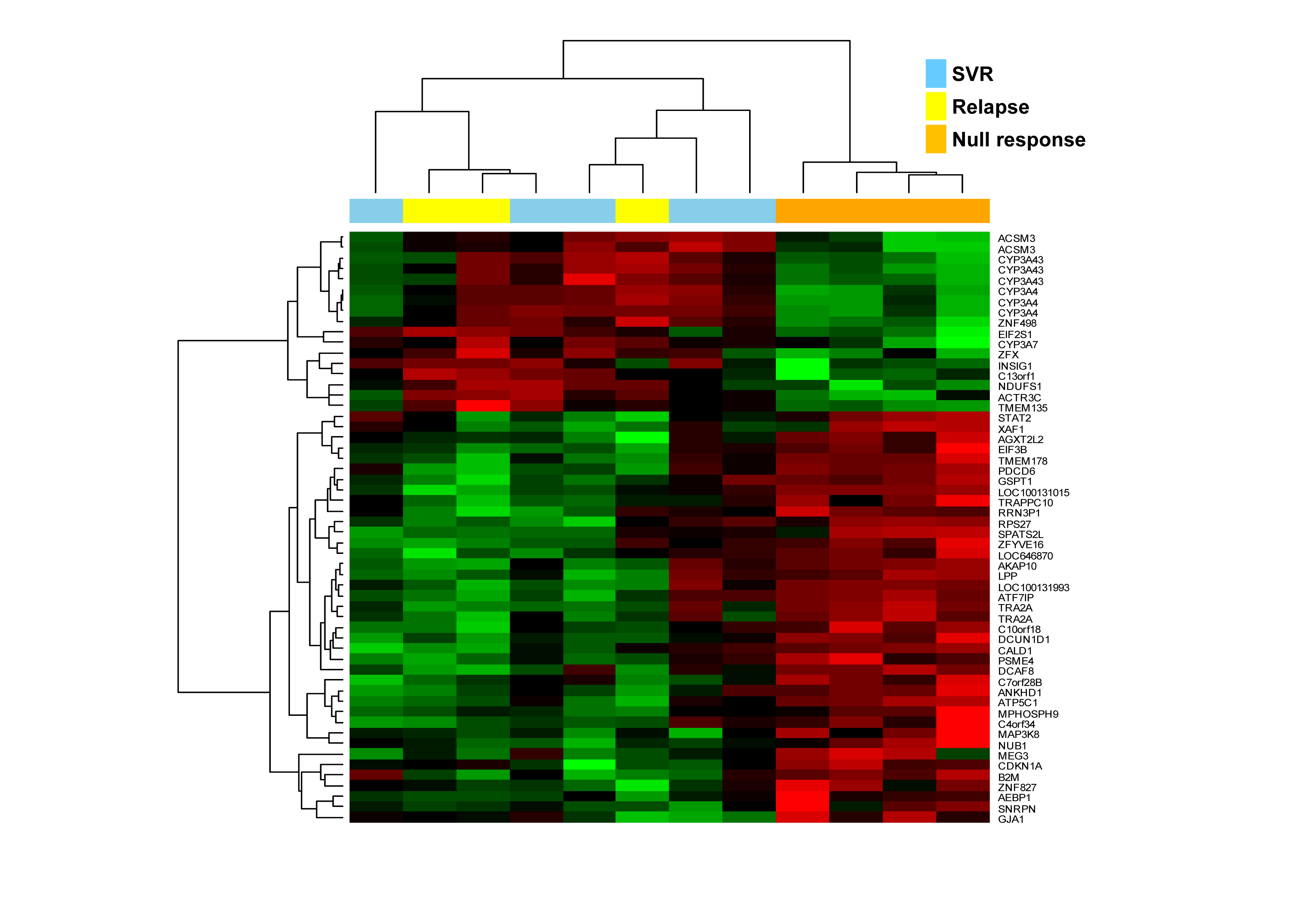

Supplement: Figure S1 — Hierarchical cluster analysis of mRNA expression using microarray analysis. Changes in mRNA expression levels are presented in graduated color patches from green (least expression) to red (most abundant expression). (TIF) [file pone.0097078.s001.tif]

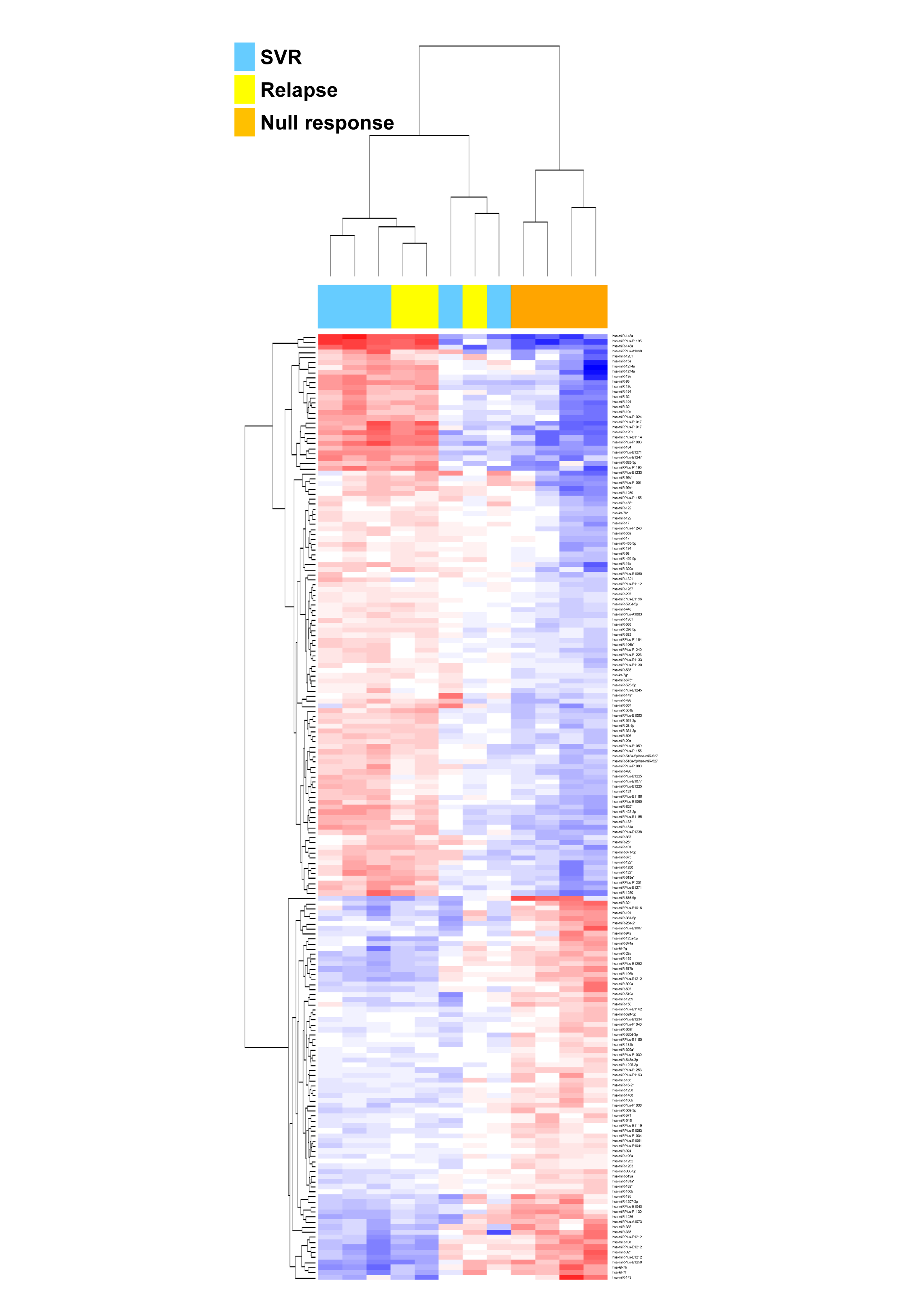

Supplement: Figure S2 — Hierarchical cluster analysis of miRNA expression using microarray analysis. Changes in gene expression are presented in graduated color patches from blue (least expression) to red (most abundant expression). (TIF) [file pone.0097078.s002.tif]

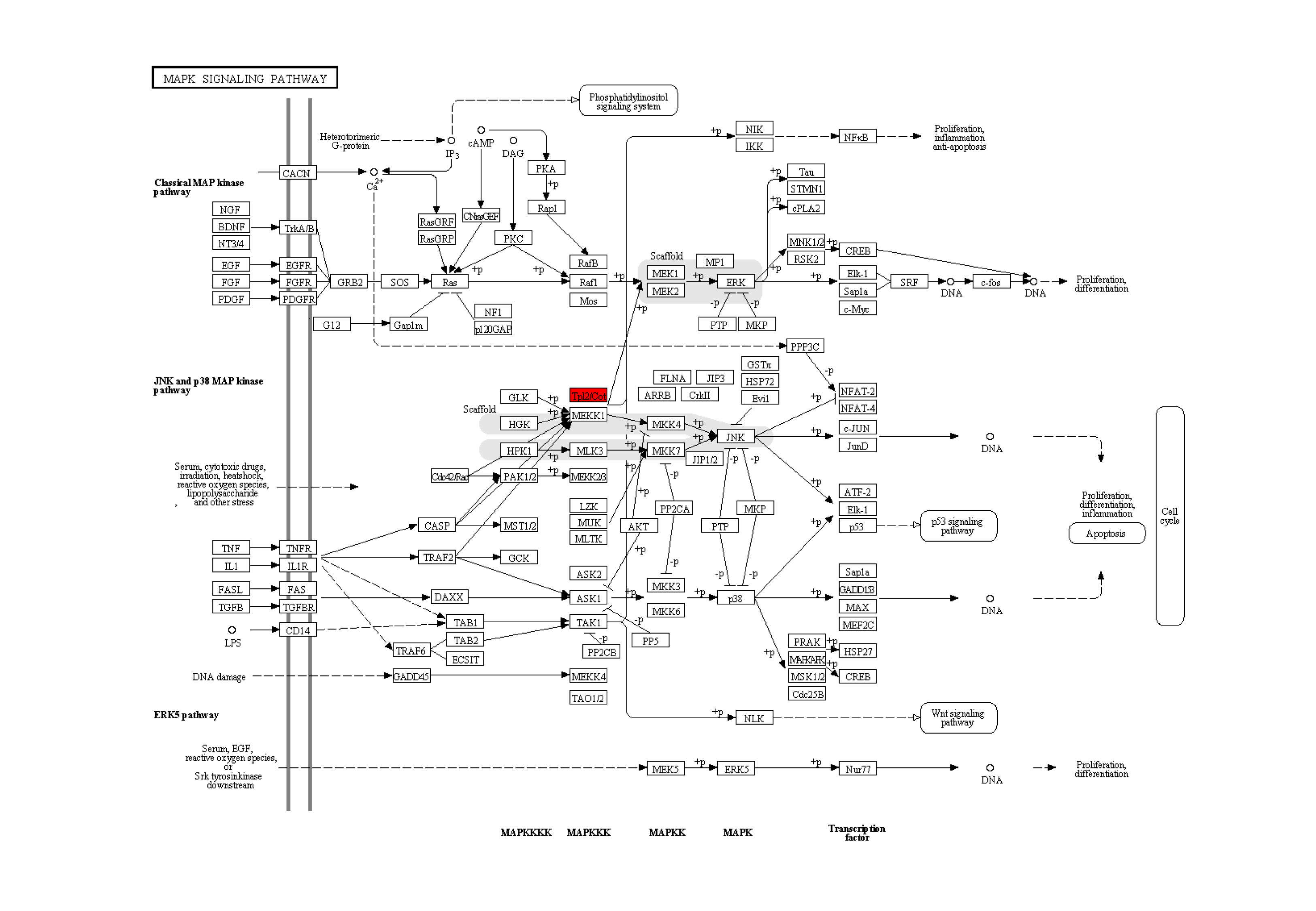

Supplement: Figure S3 — Relationship between MAP3K8 (Tpl2/Cot) and related genes in underlying gene regulatory networks. MAP3K8 (Tpl2/Cot) was integrated by Kyoto Encyclopedia of Genes and Genomes (KEGG) Pathways. MAP3K8 (Tpl2/Cot) was identified as an important node and considered to be a key regulator. (TIF) [file pone.0097078.s003.tif]

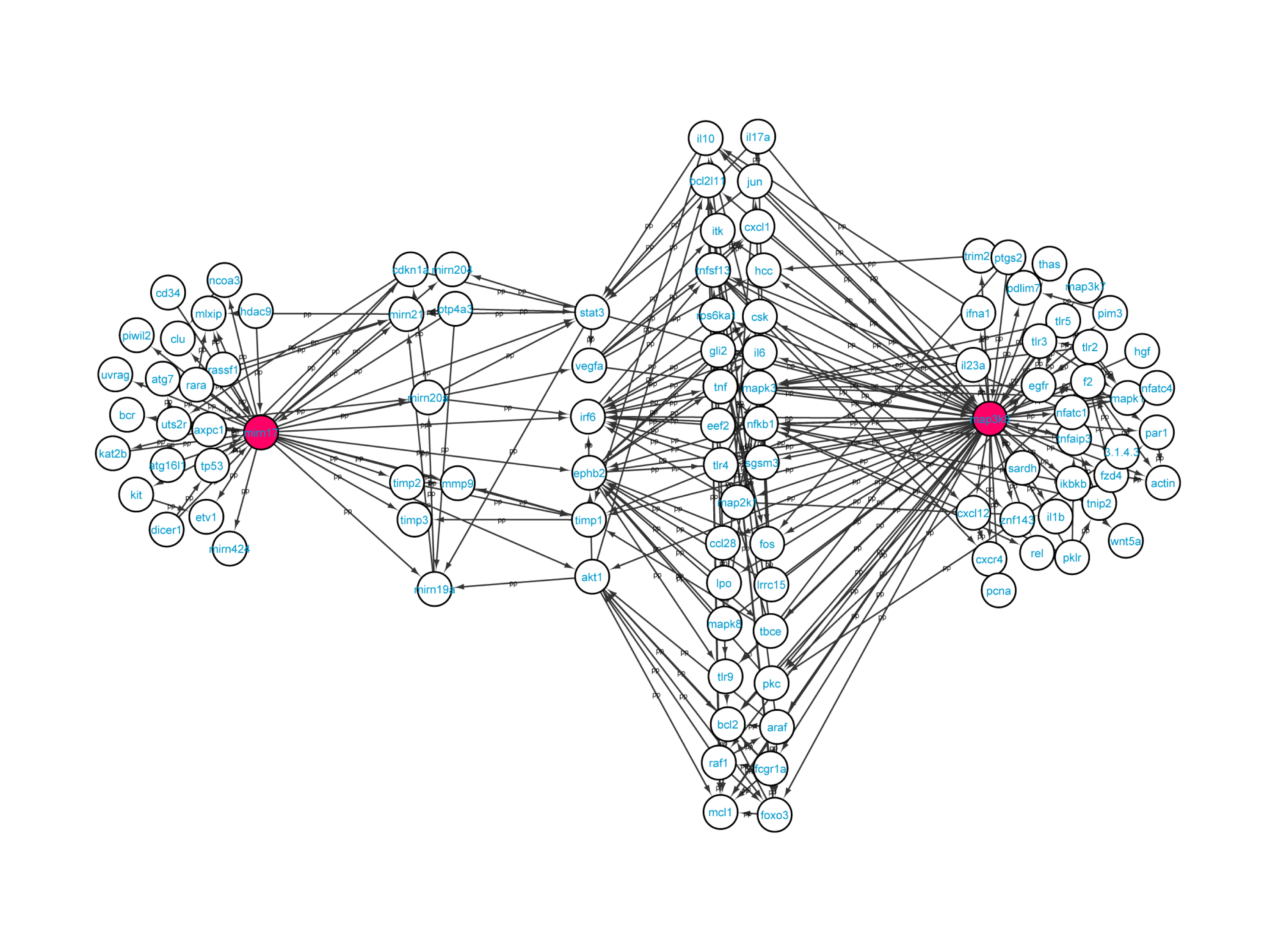

Supplement: Figure S4 — Gene networks for MAP3K8 and hsa-miR-17. MAP3K8 and hsa-miR-17 and array-independent/literature-based text-mining were integrated into the gene regulatory network analysis (Agilent Literature Search). The interaction data were visualized and analyzed by Cytoscape. MAP3K8 and its related mRNAs were associated with the miR-17 cluster family and its related miRNAs via IRF6, STAT3, AKT1, EPHB2, TIMP1, and VEGFA. (TIF) [file pone.0097078.s004.tif]

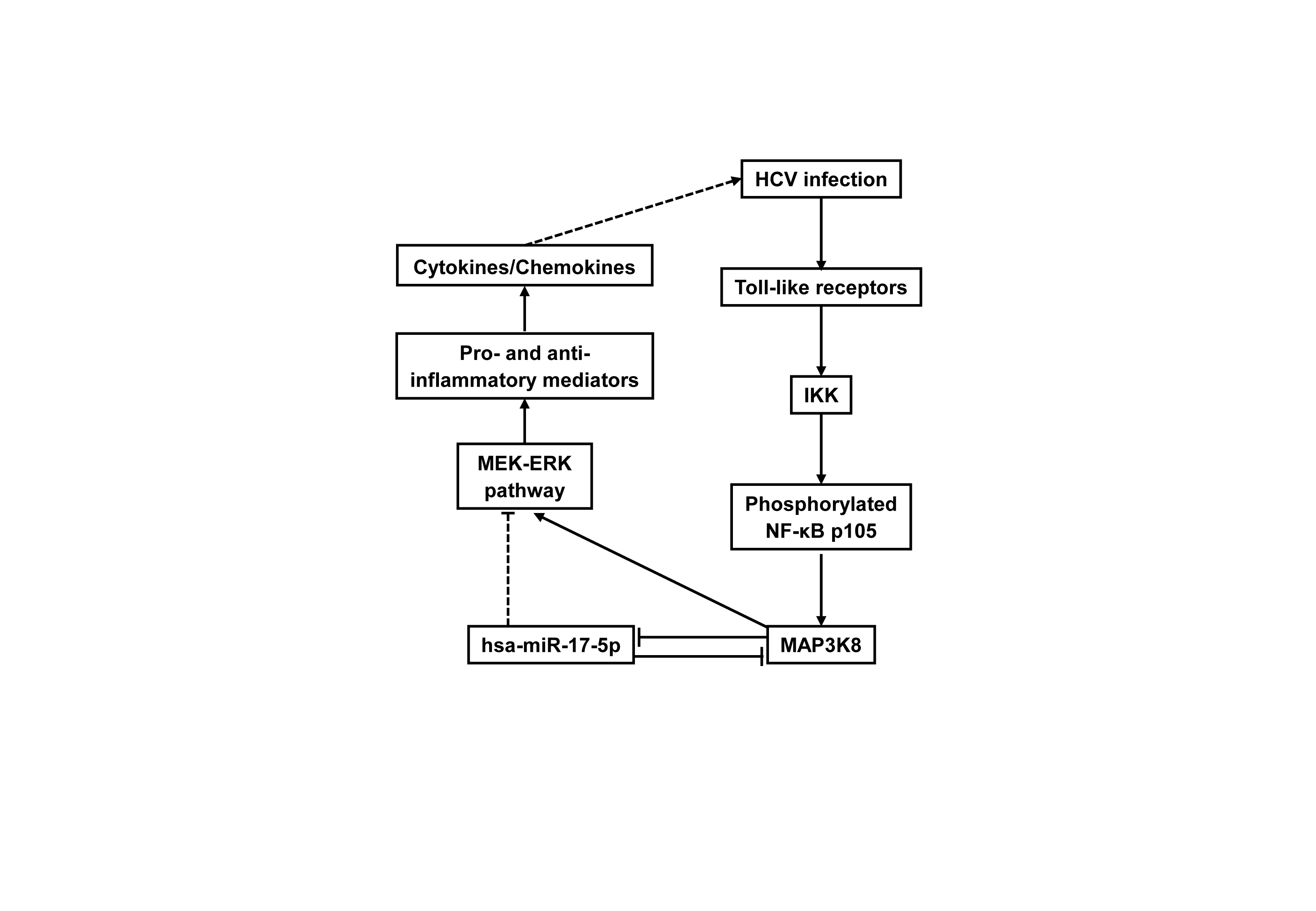

Supplement: Figure S5 — Postulated scheme for HCV replication regulated by MAP3K8 and hsa-miR-17-5p. IKK, inhibition of kappa B kinase; NF-κB, nuclear factor kappa B; MAP3K8, mitogen-activated protein kinase kinase kinase 8; MEK, MAPK/extracellular signal-regulated kinase. (TIF) [file pone.0097078.s005.tif]
