# Supplementary material for: Involvement of MAP3K8 and miR-17-5p in Poor Virologic Response to Interferon-Based Combination Therapy for Chronic Hepatitis C
Source: PLoS One. 2014 May 12;9(5):e97078. doi: 10.1371/journal.pone.0097078 (PMC4018277; doi:10.1371/journal.pone.0097078)
Supplement: Table S1 — Comparison of baseline profiles between SVRs/relapsers and null/partial responders. (DOC) [file pone.0097078.s006.doc]

**Table S1**. Comparison of baseline profiles between SVRs/relapsers and null/partial responders

| Variables | SVRs/Relapsers (*n* = 98) | Null/Partial  responders  (*n* = 32) | *p* value |
| --- | --- | --- | --- |
| Demographic data |  |  |  |
| Age (years) | 58 ± 10 | 57 ± 9 | 0.510 |
| Gender (female/male) | 45/53 | 18/14 | 0.310 |
| Body weight (kg) | 58.7 ± 11.5 | 60.5 ± 13.4 | 0.748 |
| Body mass index (kg/m^2^) | 22.2 ± 3.0 | 22.6 ± 3.3 | 0.808 |
| Laboratory tests |  |  |  |
| AST (IU/L) | 49 ± 30 | 55 ± 33 | 0.276 |
| ALT (IU/L) | 59 ± 43 | 66 ± 45 | 0.287 |
| GGT (IU/L) | 44 ± 37 | 68 ± 38 | 1.65×10^-3^ |
| Albumin (g/dL) | 4.2 ± 0.3 | 4.0 ± 0.3 | 6.66×10^-3^ |
| WBC count (×10^3^/μL) | 5.0 ± 1.5 | 4.7 ± 1.5 | 0.121 |
| Hemoglobin (g/dL) | 14.2 ± 1.3 | 14.4 ± 1.8 | 0.601 |
| Platelet count (×10^4^/μL) | 16.3 ± 5.0 | 14.6 ± 4.7 | 0.0742 |
| LDL-cholesterol (mg/dL) | 100 ± 30 | 88 ± 23 | 0.127 |
| Fasting glucose (mg/dL) | 102 ± 19 | 107 ± 25 | 0.436 |
| Liver histopathology |  |  |  |
| Stage of fibrosis: 1/2-4 | 53/45 | 10/22 | 0.0248 |
| Grade of inflammation: 1/2/3 | 68/29/1 | 20/12/0 | 0.469 |
| Host genome |  |  |  |
| *IL28B* rs8099917 TT/TG | 76/22 | 9/23 | 3.35×10^-7^ |
| *IL28B* rs12979860 CC/CT | 74/24 | 9/23 | 1.27×10^-6^ |
| *ITPA* rs1127354 CC/CA/AA | 71/24/3 | 27/5/0 | 0.174 |
| Virology |  |  |  |
| Viral load (log_10_ IU/mL) | 6.4 ± 0.6 | 6.5 ± 0.5 | 0.762 |

Data are expressed as mean ± SD or number of subjects. SVRs, sustained virological responders; AST, aspartate aminotransferase; ALT, alanine aminotransferase; GGT, gamma glutamyl transpeptidase; WBC, white blood cell; LDL, low-density lipoprotein; IL28B, interleukin 28B; ITPA, inosine triphosphatase.
